# Antibody Treatment against Angiopoietin-Like 4 Reduces Pulmonary Edema and Injury in Secondary Pneumococcal Pneumonia

**DOI:** 10.1128/mBio.02469-18

**Published:** 2019-06-04

**Authors:** Liang Li, Benjamin Jie Wei Foo, Ka Wai Kwok, Noriho Sakamoto, Hiroshi Mukae, Koichi Izumikawa, Stéphane Mandard, Jean-Pierre Quenot, Laurent Lagrost, Wooi Keong Teh, Gurjeet Singh Kohli, Pengcheng Zhu, Hyungwon Choi, Martin Lindsay Buist, Ju Ee Seet, Liang Yang, Fang He, Vincent Tak Kwong Chow, Nguan Soon Tan

**Affiliations:** a Institute of Biomedicine and Biotechnology, Shenzhen Institutes of Advanced Technology, Chinese Academy of Sciences, Nanshan, Shenzhen, China; b School of Biological Sciences, Nanyang Technological University, Singapore, Singapore; c Department of Respiratory Medicine, Unit of Translational Medicine, Nagasaki University Graduate School of Biomedical Sciences, Nagasaki, Japan; d INSERM LNC, UMR1231, Dijon, France; e University Bourgogne Franche-Comté, LNC UMR1231, Dijon, France; f LipSTIC LabEx, Fondation de Coopération Scientifique Bourgogne Franche-Comté, Dijon, France; g University Hospital of Dijon, Dijon, France; h Intensive Care Unit, University Hospital of Dijon, Dijon, France; i Singapore Centre for Environmental Life Sciences Engineering (SCELSE), Interdisciplinary Graduate School, Nanyang Technological University, Singapore, Singapore; j Singapore Centre for Environmental Life Sciences Engineering (SCELSE), Nanyang Technological University, Singapore, Singapore; k Saw Swee Hock School of Public Health, National University of Singapore, Singapore; l Department of Biomedical Engineering, Faculty of Engineering, National University of Singapore, Singapore; m Department of Pathology, National University Hospital, Singapore, Singapore; n School of Medicine, Southern University of Science and Technology, Shenzhen, China; o Institute of Preventive Veterinary Medicine, and Zhejiang Provincial Key Laboratory of Preventive Veterinary Medicine, College of Animal Sciences, Zhejiang University, Hangzhou, China; p Host and Pathogen Interactivity Laboratory, Department of Microbiology and Immunology, Yong Loo Lin School of Medicine, National University of Singapore, Singapore; q Lee Kong Chian School of Medicine, Nanyang Technological University, Singapore, Singapore; Albert Einstein College of Medicine

**Keywords:** ANGPTL4, secondary bacterial pneumonia, antibiotic resistance, host-directed immunotherapeutics, vascular permeability

## Abstract

Secondary bacterial lung infection by Streptococcus pneumoniae (S. pneumoniae) poses a serious health concern, especially in developing countries. We posit that the emergence of multiantibiotic-resistant strains will jeopardize current treatments in these regions. Deaths arising from secondary infections are more often associated with acute lung injury, a common consequence of hypercytokinemia, than with the infection *per se*. Given that secondary bacterial pneumonia often has a poor prognosis, newer approaches to improve treatment outcomes are urgently needed to reduce the high levels of morbidity and mortality. Using a sequential dual-infection mouse model of secondary bacterial lung infection, we show that host-directed therapy via immunoneutralization of the angiopoietin-like 4 c-isoform (cANGPTL4) reduced pulmonary edema and damage in infected mice. RNA sequencing analysis revealed that anti-cANGPTL4 treatment improved immune and coagulation functions and reduced internal bleeding and edema. Importantly, anti-cANGPTL4 antibody, when used concurrently with either conventional antibiotics or antipneumolysin antibody, prolonged the median survival of mice compared to monotherapy. Anti-cANGPTL4 treatment enhanced immune cell phagocytosis of bacteria while restricting excessive inflammation. This modification of immune responses improved the disease outcomes of secondary pneumococcal pneumonia. Taken together, our study emphasizes that host-directed therapeutic strategies are viable adjuncts to standard antimicrobial treatments.

## Abstract (precis)

Despite extensive global efforts, secondary bacterial pneumonia still represents a major cause of death in developing countries and is an important cause of long-term functional disability arising from lung tissue damage. Newer approaches to improving treatment outcomes are needed to reduce the significant morbidity and mortality caused by infectious diseases. Our study, using an experimental mouse model of secondary S. pneumoniae infection, shows that a multimodal treatment that concurrently targets host and pathogen factors improved lung tissue integrity and extended the median survival time of infected mice. The immunoneutralization of host protein cANGPTL4 reduced the severity of pulmonary edema and damage. We show that host-directed therapeutic strategies as well as neutralizing antibodies against pathogen virulence factors are viable adjuncts to standard antimicrobial treatments such as antibiotics. In view of their different modes of action compared to antibiotics, concurrent immunotherapies using antibodies are potentially efficacious against secondary pneumococcal pneumonia caused by antibiotic-resistant pathogens.

## INTRODUCTION

Bacterial pneumonia may present as a primary disease or as the terminal event in individuals who are already debilitated. Secondary bacterial infections resulting from an influenza infection caused over half of the deaths during the 1918 flu pandemic and remain one of the leading causes of mortality due to flu infection (1). Streptococcus pneumoniae (S. pneumoniae) is the most common pathogen responsible for secondary bacterial infections during influenza pandemics (2, 3). During the 2009 H1N1 pandemic, although antibiotics were widely used for treatment, secondary bacterial infection was detected in 55% of the fatal cases, with 26% of mortality being attributed to secondary S. pneumoniae infection (4). That value increased to 34% for patients in intensive care units (5). Current vaccines against S. pneumoniae, such as the 13-valent pneumococcal conjugate vaccine, have reduced pneumococcal infection rates (6, 7). However, the benefits of vaccination are also significantly diminished by epidemiological shifts (8). Among the six clinical trials that evaluated vaccine effectiveness against all-cause pneumonia in older adults, only one trial demonstrated a risk reduction in the vaccinated group (9). These findings underscore the importance of managing patients with influenza who may also have bacterial pneumonia.

Although antibiotics remain the mainstay of treatment for bacterial pneumonia, their broad usage has limitations and may cause certain complications. While bacteriolytic antibiotic treatment may eradicate S. pneumoniae, it causes the release of cytoplasmic virulence factors such as pneumolysin (10, 11), which is a pore-forming toxin produced by all clinical isolates of S. pneumoniae (12). This results in clinical complications even after the eradication of the pathogen (13). During a lung infection, free pneumolysin causes a disruption of the alveolus-capillary barrier, leading to pulmonary leakage and exacerbating disease severity (14). In addition, antibiotic resistance is recognized as a growing global threat. Resistance to new antibiotics is emerging at an ever-increasing pace, and at the same time, the rate of development of new antibiotics has slowed substantially. The increasing incidence of drug-resistant S. pneumoniae (DRSP) has complicated the treatment and management of various pneumococcal disease manifestations (15). Over the years, S. pneumoniae has continued to develop antibiotic resistance and poses a serious challenge to public health, particularly in Asian countries, which have among the highest rates of antibiotic resistance in the world (16, 17). In Asia, among the isolates belonging to the most prominent non-PCV7 serotype, 19A, 86.0% and 79.8% showed erythromycin resistance and multidrug resistance, respectively (16). Clearly, novel alternative countermeasures against pneumonia are urgently needed (18–20).

Many strategies that exploit components of the immune system are being actively pursued, although most are still in their infancy (21, 22). Immunotherapy with antibodies has taken a leading role in this field. In view of their different modes of action compared to antibiotics, antibodies are effective against infections with antibiotic-resistant pathogens. The synergistic use of antibodies with antibiotics can be an effective approach to managing difficult-to-treat pneumonia. High-throughput RNA sequencing of lung tissue samples from patients during the 1918 and 2009 influenza pandemics revealed that the angiopoietin-like 4 protein (ANGPTL4) was one of the most significantly upregulated mRNAs (23). We previously showed that upregulation of the C-terminal ANGPTL4 fragment (cANGPTL4) was associated with increased severity of pulmonary leakage and damage in primary influenza pneumonia (24). ANGPTL4 knockout (−/−) mice exhibit reduced pulmonary edema and improved lung tissue integrity in primary influenza pneumonia (24). These observations underscore the importance and conserved role of cANGPTL4 in the host response to infection-induced lung injury. In this study, we examined the host response to concurrent treatment of secondary bacterial pneumonia with a neutralizing anti-cANGPTL4 antibody together with antibiotics and/or a bacterial pneumolysin-targeted antibody.

## RESULTS

### Secondary bacterial infections following primary influenza compromise pulmonary integrity and augment lung edema.

**FIG 1:**
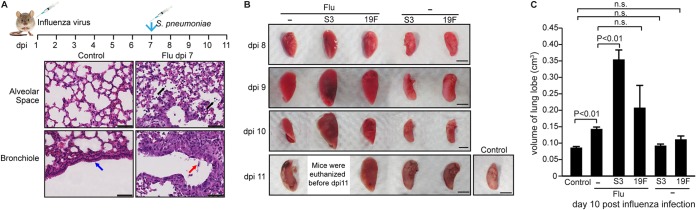
Secondary pneumococcal infection significantly increases pulmonary edema and tissue damage in a serotype-specific manner. (A) BALB/c mice were infected with a sublethal dose of the PR8 virus, followed by S. pneumoniae infection. Representative H&E images of influenza-infected lung tissue show bronchiolar epithelial damage and alveolar infiltration at 7 dpi. Influenza infection caused cell infiltration into alveolar spaces (black arrows) and bronchiolar epithelial damage (red arrow), compared to the clean alveolar spaces and intact bronchiolar epithelium (blue arrow) in control lung sections from healthy mouse lungs. Scale bar = 50 μm. (B) Representative images of infected lungs showing pulmonary edema and tissue damage. BALB/c mice were infected with a sublethal dose of the PR8 virus, followed by either serotype 3 of S. pneumoniae (Flu+S3), serotype 19F of S. pneumoniae (Flu+19F), or sterile PBS (Flu) at 7 dpi. Mice infected directly with S3 or 19F of S. pneumoniae or sterile PBS (control) were included for comparison. *n* = 3 independent experiments with 5 mice per time point for each experimental group. Infection-induced edema was evident as early as 9 dpi. Scale bar = 5 mm. (C) Graph showing the mean volume (cm^3^) of lung samples from infected and control mice in panel B.

Among the clinical cases of influenza infection, the associated secondary bacterial pneumonia is a leading cause of death (1), with S. pneumoniae being the most common causative bacterium (2, 3). The most prevalent serotype encountered in the Asian population is 19F, whereas serotype 3 is more virulent (16, 25). To address the possible role of cANGPTL4 in secondary pneumococcal pneumonia, we developed a sequential dual-infection mouse model, mimicking the clinical scenario wherein patients with a primary influenza infection are susceptible to secondary pneumococcal pneumonia. Mice were intratracheally infected with the influenza virus on day 0 and subsequently infected intratracheally with either S. pneumoniae serotype 3 (S3) or 19F at 7 days post-influenza infection (dpi) (Fig. 1A).

Next, we examined the host expression profile and the pathological phenotype of the lung tissues in this sequential dual-infection mouse model. The bronchiolar epithelium and alveolar tissues were infiltrated with immune cells and damaged by the influenza virus infection at 7 dpi prior to bacterial challenge (Fig. 1A). From 8 to 11 dpi, lungs were harvested daily from mice infected by the influenza virus (Flu), bacteria (S3 or 19F), or influenza with bacterial superinfection (Flu+S3 or Flu+19F). To assess the extent of edema in the infected lungs, the volumes of the harvested lung lobes were measured. Lung lobes with more edema display larger volumes due to fluid infiltration from the circulation (Fig. 1B). The lungs of the primary influenza-infected mice showed a small, but significant, 1.5-fold increase in lung volume, while primary bacterial 19F- and S3-infected lungs exhibited no significant change in lung volume compared with the sham-infected lungs (Fig. 1C). Secondary bacterial infection resulted in significantly greater edema than primary influenza infection. They also showed clear pneumococcal serotype-specific patterns (Fig. 1B and C). Mice infected with influenza and serotype 3 of S. pneumoniae (Flu+S3) reached the criteria for euthanasia (i.e., at least 30% loss of their body weight) before 11 dpi (Fig. 1B). The lungs of mice infected with Flu+S3 displayed a significant ≥2.0-fold increase in lung lobe volume compared with sham-infected lungs (Fig. 1C). S. pneumoniae cells were detected in the pulmonary epithelium and alveolar spaces, as determined by fluorescence *in situ* hybridization (FISH). The fluid-filled alveolar spaces may encourage bacterial growth, and S. pneumoniae cells were detected in abundance in those spaces (see Fig. S1A in the supplemental material).

10.1128/mBio.02469-18.1 FIG S1 Primary influenza infection augments pulmonary damage associated with secondary bacterial infections. (A) Fluorescence *in situ* hybridization (FISH) of infected mouse lung tissue to identify the distribution of bacteria. Scale bar = 10 μm. (B) Representative hematoxylin and eosin (H&E) staining on harvested lung tissue showing tissue damage morphology. Scale bar = 100 μm. (C) Percentage of intact alveoli in lungs from mice with the indicated infection relative to the mock infection control. The mock infection control was set at 100%. Ctrl, mock infection with sterile PBS; Flu, influenza infection alone; S3, S. pneumoniae serotype 3 infection alone; 19F, S. pneumoniae serotype 19F infection alone; Flu+S3, influenza infection followed by infection with S. pneumoniae serotype 3; Flu+19F, influenza infection followed by infection with S. pneumoniae serotype 19F. *n* = 5 mice per time point for each experimental group. Download FIG S1, TIF file, 2.8 MB. Copyright © 2019 Li et al. 2019 Li et al. This content is distributed under the terms of the Creative Commons Attribution 4.0 International license.

Histological analysis of the lungs revealed significantly greater lung tissue damage in secondary pneumococcal infections compared with primary influenza infection alone (Fig. S1B). Regions of lung tissue from Flu+S3-superinfected mice lost the basic structure of the alveolar walls and were replaced with excessive fluid arising from severe lung edema. Compared to the Flu+S3 mice, the Flu+19F-superinfected mice displayed better preserved alveolar structures, although there was still significantly more fluid infiltration in the alveolar spaces compared to mice with influenza infection alone. More intra-alveolar erythrocytes and immune cells infiltrated the lung parenchyma in influenza-infected mice than in primary bacterial pneumonia-infected mice. The lungs of primary bacterial pneumonia-infected mice exhibited pulmonary tissue morphology similar to that of healthy control lungs, suggesting that primary bacterial infection did not cause noticeable tissue damage. The trends in lung tissue damage among the different experimental groups were indicated by the morphology of the alveolar structures and the percentage of the intact alveoli compared to healthy lung lobes. Sham-infected, S3-infected, and 19F-infected mice showed uncompromised alveolar space integrity, whereas secondary pneumococcal pneumonia-infected mice suffered more substantial lung tissue injury than influenza-infected mice. Secondary pneumococcal pneumonia-infected mice revealed distinct serotype specificity, with Flu+S3 mice suffering more severe edema and loss of alveolar structures than Flu+19F mice (Fig. S1C). Taken together, secondary pneumococcal infection following primary influenza infection significantly increased the severity of lung edema and pulmonary tissue damage in a serotype-specific manner.

### cANGPTL4 expression is upregulated in the lung tissues of mice with secondary pneumococcal pneumonia.

**FIG 2:**
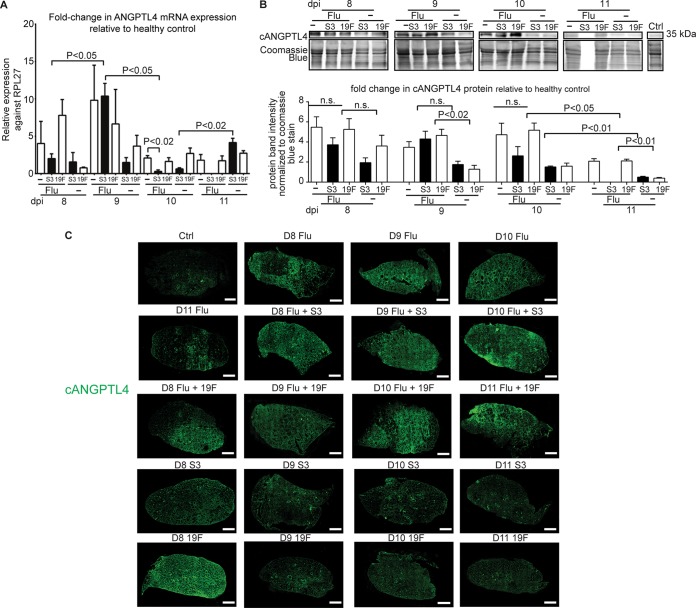
ANGPTL4 mRNA and protein are significantly upregulated in secondary pneumococcal pneumonia-infected mouse lung tissue. (A) Relative ANGPTL4 mRNA expression in lung tissues infected with the influenza virus and/or S. pneumoniae. rRNA L27 (RPL27) served as the reference or housekeeping gene. Data are represented as the mean ± standard deviation (SD) from at least three independent experiments. (B) Relative cANGPTL4 protein expression in lung tissues infected with the influenza virus and/or S. pneumoniae. Coomassie staining of immunoblots served as loading and transfer controls. Loading controls for the immunoblot analyses were from the same samples. Data are represented as the mean ± SD from at least three independent experiments. (C) Representative immunofluorescent staining for cANGPTL4 in lungs from control and infected mice. Scale bar = 1 mm.

In correlation with the severity of lung damage, quantitative PCR (qPCR) analysis showed that ANGPTL4 mRNA levels peaked at 9 dpi in lungs infected with Flu (9-fold), 19F (4-fold), S3 (2-fold), Flu+19F (7.5-fold), and Flu+S3 (10-fold) compared to sham-infected lungs (Fig. 2A). Immunoblot analysis revealed elevated levels of cANGPTL4 protein from 8 to 10 dpi across all of the diseased lungs compared to the healthy lungs (Fig. 2B). Immunofluorescence staining of the lung tissue revealed a widespread distribution of cANGPTL4 in mice with primary influenza and secondary bacterial pneumonia (Flu+S3 and Flu+19F) at all examined time points (Fig. 2C). In contrast, primary bacterial pneumonia-infected lungs showed cANGPTL4 immunostaining patterns that were similar to those in healthy control lung sections, in which cANGPTL4 was restricted to the bronchiolar structures (Fig. 2C). Thus, elevated cANGPTL4 expression levels correlate well with pulmonary damage in secondary bacterial pneumonia.

### Deficiency in host cANGPTL4 reduces pulmonary edema and protects lung tissue integrity in mice with secondary pneumococcal pneumonia.

**FIG 3:**
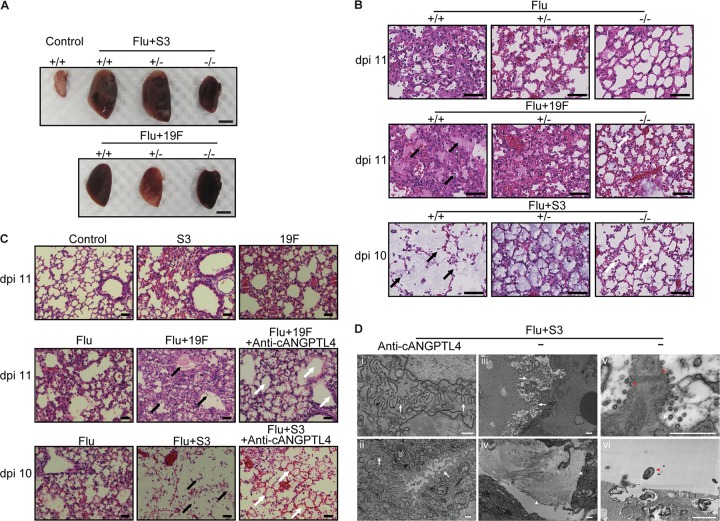
Deficiency in the cANGPTL4 protein reduces pulmonary edema and protects lung tissue integrity in infected mice. (A) Representative image of lung harvested at 11 dpi from ANGPTL4 knockout (−/−), heterozygous (+/−), and wild-type (+/+) mice infected with influenza virus and different pneumococcal serotypes (S3 and 19F). Scale bar = 5 mm. *n* = 3 independent experiments with 5 mice per time point for each experimental group. (B) Representative H&E images of the indicated primary and secondary pneumococcal infections of lungs from ANGPTL4 knockout (−/−), heterozygous (+/−), and wild-type (+/+) mice. In Flu+19F-infected mice, the ANGPTL4 knockout group showed significant reduction of immune cell infiltration and edema compared with their wild-type counterparts (black versus white arrows); In Flu+S3-infected mice, the wild-type group showed vanished alveolar structures with severe edema, while the ANGPTL4 knockout group preserved the alveolar structures with reduced edema (black versus white arrows). Scale bar = 50 μm. (C) Infected wild-type mice treated with the anti-cANGPTL4 antibody compared with untreated mice. In the Flu+19F-infected mice, anti-cANGPTL4 treatment induced improved bronchiolar epithelial integrity and reduced infiltration (black versus white arrows). In the Flu+S3-infected mice, anti-cANGPTL4 treatment better preserved the structure of the alveolus (white arrow), compared to the diminished alveolar structures due to severe edema in the lungs of the Flu+S3 group of mice (black arrow). Scale bar = 50 μm. (D) Representative transmission electron microscopy images of lungs from mice with a secondary infection of serotype 3 that were administered the anti-cANGPTL4 antibody (i and ii) or control IgG (iii and iv). White arrows and arrowheads indicate cell-cell junctions and collagen fibrils, respectively. Viral budding (v) is visible at the damaged cell boundary, and bacteria are visible in control vehicle-treated mice with a secondary infection of serotype 3 (vi). Red arrowheads indicate viral budding and bacteria. Scale bar = 500 nm.

The abundance of the cANGPTL4 protein in secondary bacterial pneumonia-infected mouse lung tissue suggests an important role for cANGPTL4 in infection-induced pulmonary damage. To this end, we examined its impact on lung edema and tissue integrity during secondary bacterial pneumonia using ANGPTL4 knockout mice (ANGPTL4^−/−^) (Fig. 3A and B) and immunoneutralization of cANGPTL4 (Fig. 3C and D). ANGPTL4^−/−^ mice with secondary Flu+S3 and Flu+19F infections exhibited less pulmonary edema than their ANGPTL4^+/+^ counterparts (Fig. 3A and B). As indicated by the arrows in Fig. 3B, Flu+19F-infected ANGPTL4^+/+^ mouse lung tissue was characterized by extensive edema, bleeding, and immune cell infiltration in the alveolar spaces, whereas ANGPTL4^−/−^ mice with Flu+19F infection showed better-preserved integrity of the alveolar spaces. Among the Flu+S3-infected mice, ANGPTL4^+/+^ mouse lung tissue featured severe edema that destroyed the alveolar structures, leaving diminished alveolar walls, in contrast to ANGPTL4^−/−^ mice, which had preserved alveolar structures and less edema (Fig. 3B). In ANGPTL4^+/−^ mice, the improvement in lung tissue integrity was less obvious (Fig. 3A and B). We observed an improvement in lung tissue integrity in ANGPTL4^−/−^ mice compared with their wild-type counterparts for both Flu+S3 and Flu+19F superinfections, as indicated by the higher percentage of intact alveolar spaces (see Fig. S2A in the supplemental material).

10.1128/mBio.02469-18.2 FIG S2 Effects of anti-cANGPTL4 treatment in mice during secondary pneumococcal pneumonia. (A) Percentage of intact alveoli in lungs from mice with the indicated infection relative to the mock infection control. The mock infection control was set at 100%. Flu, influenza infection alone; Flu+S3, influenza infection followed by infection with S. pneumoniae serotype 3; Flu+19F, influenza infection followed by infection with S. pneumoniae serotype 19F; KO, ANGPTL4^−/−^ knockout mice; WT, ANGPTL4^+/+^ mice; Ab, anti-cANGPTL4 antibody treatment; IgG, control IgG treatment. *n* = 5 mice per time point for each experimental group. (B) Gene Ontology of differentially expressed genes in lung tissues of mice with secondary bacterial pneumonia treated with anti-cANGPTL4 antibody compared to control IgG. *n* = 5 mice were used for each experimental group. Values are shown in log_2_. Download FIG S2, TIF file, 1.6 MB. Copyright © 2019 Li et al. 2019 Li et al. This content is distributed under the terms of the Creative Commons Attribution 4.0 International license.

To confirm the results observed in ANGPTL4 knockout mice and to explore the therapeutic potential of anti-cANGPTL4 treatment, we next studied the effects of an cANGPTL4 deficiency caused by the injection of an anti-cANGPTL4 monoclonal antibody (MAb) into secondary pneumococcal pneumonia-infected mice. Infected ANGPTL4^+/+^ mice treated with anti-cANGPTL4 MAb were protected against infection-associated lung damage, with better lung tissue integrity and reduced edema compared to infected but untreated ANGPTL4^+/+^ mice, thus confirming the findings of the ANGPTL4 knockout experiments (Fig. 3C). As indicated by the higher percentage of intact alveolar spaces, we observed an improvement in lung tissue integrity in anti-cANGPTL4 MAb-treated mice compared with control IgG-treated mice for both Flu+S3 and Flu+19F superinfections (Fig. S2A). Electron micrographs further confirmed that anti-cANGPTL4 MAb treatment improved the integrity of intercellular tight junctions between lung epithelial cells compared to the IgG control treatment (Fig. 3D, panels i to iii). In contrast, the lungs of control IgG-treated mice showed increased fibrosis and bacterial spread (Fig. 3D, panels iv to vi). RNA sequencing analysis revealed that anti-cANGPTL4 MAb treatment improved immune function against secondary bacterial infection (Flu+S3), as well as coagulation to reduce bleeding and edema in the lungs (Fig. S2B). Hence, an cANGPTL4 deficiency reduces pulmonary edema and protects lung tissue integrity, suggesting that the use of anti-cANGPTL4 therapy during secondary bacterial pneumonia may be beneficial.

### Synergistic actions of antibodies with antibiotics mitigate pulmonary tissue damage and edema and prolong the survival of mice with secondary bacterial pneumonia.

**FIG 4:**
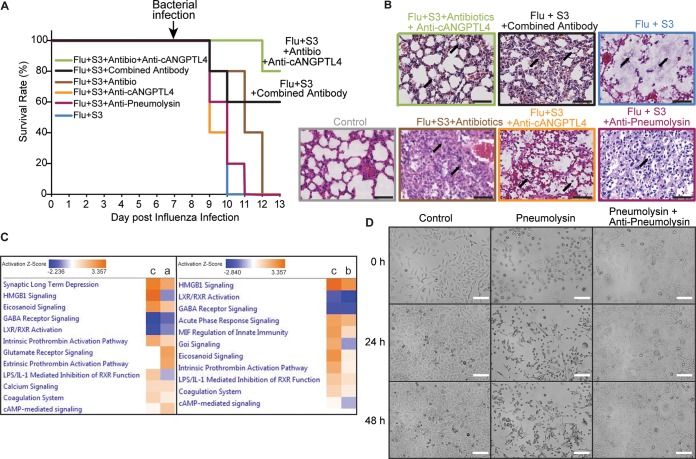
Cotreatment with antibiotics and specific antibodies ameliorates lung tissue damage and prolongs the survival of infected mice. (A) A Kaplan-Meier survival plot shows the percentage of survival of mice given the various indicated treatments. Five mice were used for each experimental group. (B) Representative H&E images of lungs from mice administered the various indicated treatments. Combined treatment with antibiotics and anti-cANGPTL4 antibody or with anti-cANGPTL4 antibody and antipneumolysin antibody further improved the lung tissue integrity from single treatment with either antibody or antibiotics. Arrows indicate edema, infiltration of immune cells, and tissue damage patterns. Scale bar = 50 μm. (C) Comparative Gene Ontology results analyzed from RNA sequencing data of lung tissues from infected mice (Flu+S3) administered moxifloxacin (a), anti-cANGPTL4 antibody (b), and a combined treatment with antibiotics and antibodies (c). (D) Representative bright-field images of A549 human alveolar epithelial cells cultured with mock IgG treatment (control), pneumolysin, or pneumolysin with an antipneumolysin antibody at the indicated times posttreatment. Cells were tracked for 48 h using the JuLi Stage system for live imaging. *n* = 4 independent experiments. Scale bar = 50 μm.

Antibiotics constitute the first-line therapy for bacterial pneumonia. To complement current clinical practice, secondary bacterial pneumonia-infected mice were treated with anti-cANGPTL4 MAb and moxifloxacin, a commonly used antibiotic effective against respiratory infections. The combined treatment significantly prolonged the median survival time of infected mice (80%) compared to zero survival of mice receiving the control IgG treatment or moxifloxacin alone (Fig. 4A; see Fig. S3A in the supplemental material). Moreover, this combined treatment better protected alveolar integrity and reduced residual fluid in the alveolar spaces, thus indicating its efficacy in ameliorating lung edema, diminishing tissue damage, and prolonging survival time (Fig. 4B; Fig. S3B and C). As indicated in Fig. 4B and quantified in Fig. S3C, Flu+S3 superinfection destroyed the alveolar structures and caused severe edema in the lungs of infected wild-type mice. Antibiotic treatment alone protected the alveolar structures but could not clear the edema and infiltration of immune cells. Although the anti-cANGPTL4 or antipneumolysin antibody treatments alone improved lung tissue integrity and reduced edema, these improvements were not as significant as those observed when using a combined treatment with antibiotics and anti-cANGPTL4 MAb or with anti-cANGPTL4 and antipneumolysin antibodies (Fig. 4B; Fig. S3C). The efficacy of a combined approach was further confirmed by marked improvements in the mouse survival rate and lung tissue integrity in treatment groups of wild-type mice receiving antibiotics, anti-cANGPTL4, and antipneumolysin MAb. Similar trends were observed in ANGPTL4^−/−^ mice receiving antibiotic and antipneumolysin treatment (Fig. S3).

10.1128/mBio.02469-18.3 FIG S3 Effects of combined treatments in mice during secondary pneumococcal pneumonia. (A) A Kaplan-Meier survival plot shows the percentage of survival of ANGPTL4^+/+^ and ANGPTL4^−/−^ mice given the various indicated treatments. Five mice were used for each experimental group. (B) Representative H&E images of lungs from mice administered the various indicated treatments. Scale bar = 100 μm. (C) Percentage of intact alveoli in lungs from mice given the various indicated treatments compared to control. *n* = 5 mice per time point for each experimental group. Download FIG S3, TIF file, 1.9 MB. Copyright © 2019 Li et al. 2019 Li et al. This content is distributed under the terms of the Creative Commons Attribution 4.0 International license.

Next, we performed transcriptomic analysis of the lung tissues to elucidate the detailed host response factors to secondary pneumococcal pneumonia during the various treatment modalities (Fig. 4C). Compared with antibiotic treatment alone, our analysis indicated that the anti-cANGPTL4 MAb treatment combined with an antibiotic notably improved the immune responses against bacterial infection as well as coagulation function, thus attenuating intra-alveolar hemorrhage and edema in the lungs of secondary bacterial pneumonia-infected mice. For example, this combination treatment yielded a 3-fold increase in activation of the coagulation system pathway compared to MAb treatment alone (Fig. 4C).

### Immunoneutralization of pneumococcal pneumolysin abrogates its pore-forming action on alveolar epithelium.

Antibiotic resistance among clinical strains of S. pneumoniae underscores the urgency for alternative treatment strategies. Multimodal host- and pathogen-directed immunotherapy is a feasible option. Thus, we employed the experimental dual-infection mouse model to explore concurrent immunotherapy for secondary bacterial pneumonia. The host response protein cANGPTL4 and bacterial virulence factor pneumolysin were targeted using cognate neutralizing antibodies. The antipneumolysin antibody mitigated the pore-forming action of pneumolysin in human alveolar epithelial cells and reduced tissue damage. This effect persisted for 48 h (Fig. 4D). The concurrent antibiotic and antibody treatment significantly improved lung tissue integrity and, importantly, extended the median survival time of mice with secondary bacterial pneumonia compared to treatment with either the antibiotic or a single antibody (Fig. 4A and B). Taken together, these observations highlight that host-directed therapeutic anti-cANGPTL4 MAb can complement pathogen-directed treatment, such as conventional antibiotics or a novel antipneumolysin antibody, to enhance lung tissue integrity and augment host survival during secondary pneumococcal pneumonia. The improved tissue integrity and considerably longer median survival time suggest that a multimodal treatment approach targeting both host and pathogen factors can be highly efficacious against DRSP infection.

### Immunotherapy against cANGPTL4 enhances host immune responses against secondary pneumococcal pneumonia.

**FIG 5:**
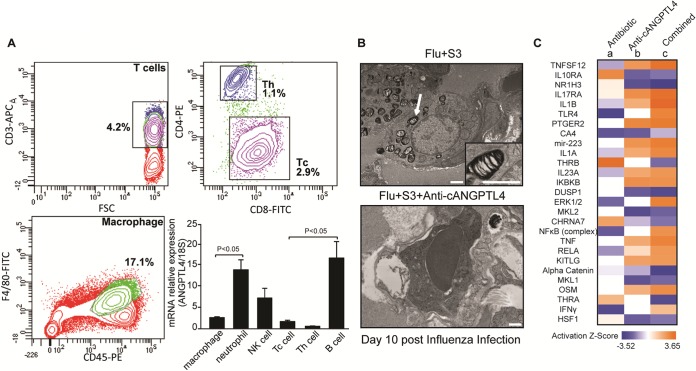
cANGPTL4 immunoneutralization activates immune defenses against bacterial infection. (A) Using mouse lung tissue harvested from mice with a secondary infection of serotype 3 on day 9 post-influenza infection, representative FACS plots are shown for the major groups of immune cells sorted according to their surface markers. The immune cell groups include macrophages (F4/80^+^ and CD45^+^), neutrophils (Ly6G^+^), natural killer cells (NK1.1^+^), cytotoxic T cells (Tc; CD3^+^ and CD8^+^), T helper cells (Th; CD3^+^ and CD4^+^), and B cells (CD19^+^). The graph shows the different relative expression levels of ANGPTL4 as determined by qPCR, with 18S rRNA serving as a reference housekeeping gene. *n* = 3 independent experiments. (B) Representative transmission electron microscopy images of immune cells from lungs of secondary bacterial pneumonia-infected mice administered the control IgG treatment (Flu+S3) and the anti-cANGPTL4 antibody treatment (Flu+S3+anti-cANGPTL4). White arrows indicate semidigested bacteria. The inset shows a higher-magnification view of a semidigested bacterium inside a lymphocyte. Scale bar = 500 nm. (C) Heat map profile indicating the expression of specific upstream gene regulators in lung tissues from infected mice administered moxifloxacin (a), anti-cANGPTL4 antibody (b), and a combined treatment with the antibiotic and antibody (c) as in Fig. 4C.

To gain further insights into the host responses to secondary pneumococcal infection and the various treatments, we analyzed the immune cells and gene expression of the infected mouse lungs. qPCR on fluorescence-activated cell sorter (FACS)-sorted major immune cell groups revealed that macrophages, neutrophils, natural killer (NK) cells, cytotoxic T cells, and B cells expressed ANGPTL4 at various levels during secondary pneumococcal pneumonia (Fig. 5A). In Flu+S3-infected mouse lungs, semidigested bacteria appeared within the lymphocyte cytoplasm, while the antibody-mediated neutralization of cANGPTL4 augmented the ability of macrophages to phagocytose and fully digest the bacteria (Fig. 5B). The improved phagocytosis and bacterial digestion suggest that anti-cANGPTL4 treatment enhances host immune function.

Next, we further interrogated the transcriptome of lung tissues to understand the detailed host response factors to secondary pneumococcal infection and the various treatments. Our analysis showed that the anti-cANGPTL4 antibody treatment, either alone or in combination with antibiotic treatment, increased the expression of host immune defense-related genes (such as those coding for interleukin-1β [IL-1β], Toll-like receptor 4 [TLR4], NF-κB, tumor necrosis factor [TNF], and gamma interferon [IFN-γ]), while decreasing the expression of anti-inflammatory genes (such as *IL10RA*) (Fig. 5C). Interestingly, the combined treatment using anti-cANGPTL4 and an antibiotic generated a greater enhancement of gene expression than the anti-cANGPTL4 treatment alone, whereas antibiotic treatment alone produced the opposite effect in terms of the regulation of these genes (Fig. 5C). Given that an insufficient host immune response is a major contributory factor to the lethal outcome of secondary bacterial pneumonia, the apparently positive effects of anti-cANGPTL4 therapy on the immune response could be considered beneficial to the host’s defense against bacterial infection (1) and are consistent with the improved phagocytosis and bacterial digestion (Fig. 5B).

### Elevated cANGPTL4 protein levels in the bronchoalveolar lavage fluid of patients with pneumonia.

**FIG 6:**
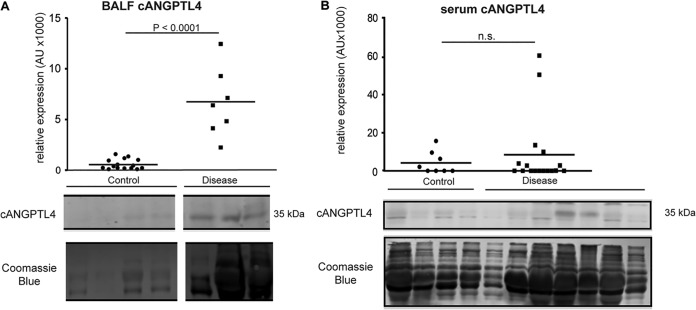
Elevated cANGPTL4 protein in the bronchoalveolar lavage fluid of patients with pneumonia. Shown are graphs depicting the relative expression of the C-terminal isoform of ANGPTL4 protein (cANGPTL4) in (A) BALF samples (control, *n* = 16; ARDS, *n* = 7) and (B) serum samples (control, *n* = 8; pneumonia, *n* = 17) from control healthy volunteers and patients with lung disease. n.s., no statistical significance. Representative images of Western blots are shown for BALF and serum samples.

All clinical isolates of S. pneumoniae secrete toxic pore-forming pneumolysin. This supports a potential broad application of an antipneumolysin antibody in new treatments against DRSP infection (12). Similarly, to underscore the potential use of anti-cANGPTL4 therapy in pulmonary diseases, we examined the protein level of cANGPTL4 in bronchoalveolar lavage fluid (BALF) from patients with acute respiratory distress syndrome (ARDS; *n* = 7). Compared with healthy volunteers (control; *n* = 16), the ARDS BALF samples showed significantly higher cANGPTL4 protein levels (∼15-fold), as detected by immunoblotting (Fig. 6A). However, this marked increase in cANGPTL4 was not observed in serum samples from pneumonia patients (*n* = 17) compared with non-pneumonia patients (control; *n* = 8) (Fig. 6B). This finding indicates that the cANGPTL4 protein was elevated in the pulmonary fluid of patients with severe pneumonia, whereas its levels in the blood circulation may be influenced by other factors. It also supports the potential utility of cANGPTL4 as a biomarker and therapeutic target for the diagnosis and treatment of infection-associated lung injury.

## DISCUSSION

S. pneumoniae is a major cause of acute pneumonia and is the most commonly identified pathogen in secondary bacterial lung infection (4). The increasing prevalence of multidrug-resistant S. pneumoniae also poses a serious public health concern worldwide, particularly in Asian countries (4, 16, 26). Secondary bacterial pneumonia presents a unique set of host responses and challenges. As this is a rapidly progressing infection, often with a poor prognosis, newer approaches for improving treatment outcomes are needed to reduce its high morbidity and mortality. Few studies have examined the effect of antibody-based immunotherapy on secondary pneumococcal pneumonia. In this study, a multimodal treatment that concurrently targets the host and pathogen factors in an experimental mouse model of secondary S. pneumoniae infection was shown to significantly improve lung tissue integrity and extend the median survival time of infected mice. Immunoneutralization of the host protein cANGPTL4 significantly diminished the severity of pulmonary edema and damage when combined with pathogen-directed therapies, such as antibiotics or antipneumolysin MAb, which attenuated bacterial replication or toxicity, ultimately considerably prolonged the survival of infected mice. Effects of anti-cANGPTL4 antibody treatment in secondary bacterial infections were also confirmed using ANGPTL4^−/−^ mice.

Complications or death arising from influenza and secondary pneumonia are often associated with hyperinduction of proinflammatory cytokines and chemokines, a phenomenon also known as a “cytokine storm” or hypercytokinemia (27–30). More patients die from ARDS, a common consequence of hypercytokinemia, than from the infection *per se* (31). Pathogen-induced lung injury can manifest as mild to severe ARDS, as seen with the severe acute respiratory syndrome (SARS) coronavirus, influenza virus, and secondary bacterial pneumococcal infections (1, 31, 32). Although inflammatory processes constitute important treatment targets (32–34), anti-inflammatory treatments can inhibit the immune functions that mediate pathogen clearance. A compromised host immune response during influenza infection renders the host more vulnerable to secondary bacterial infection. This is particularly crucial since secondary bacterial infection is usually facilitated by the influenza virus, which impairs host immune responses and alters cytokine production, thus favoring bacterial superinfection and greatly increasing host morbidity and mortality (1, 32, 35–38). For example, treatments targeting proinflammatory cytokines, reactive oxygen species (ROS), and reactive nitrogen species (RNS) come with a risk of enhancing pathogen replication (39, 40). Ideally, potential treatment regimens should minimize the tissue damage caused by inflammation and facilitate recovery without interfering with the host’s antiviral or antibacterial responses.

Both host and bacterial factors contribute to severe tissue damage in pneumonia, thus offering critical targets for therapy development (41, 42). The therapeutic use of passive antibodies has been well established for several primary viral infections (43–45). Similarly, the passive immunization afforded by antibodies to the pathogenic virulence factor pneumolysin can enhance the survival rate of mice with primary bacterial pneumonia (13). The pneumolysin gene has very limited variability and is therefore a prime pathogen target (46). Our antipneumolysin MAb targets the oligomerization domain, which is conserved across strains—i.e., it is not strain-specific, unlike previously reported antipneumolysin antibodies (13). Recent insights into pathogen-host interactions and host innate immune responses have also led to the identification and development of a wide range of host-directed therapies with different mechanisms of action. One such approach is the targeting of vascular leakage to combat respiratory diseases (24, 47–49). Recent studies have identified cANGPTL4 as a key player in tissue leakiness (24, 50, 51). Primary influenza pneumonia and lipopolysaccharide-induced acute lung injury are associated with an increased expression of cANGPTL4 (23, 24, 48). Influenza-infected cANGPTL4-deficient mice exhibit reduced lung tissue leakiness and damage compared to mice with functioning cANGPTL4 (24). Similarly, ANGPTL4 suppression by small interfering RNA (siRNA) also protects mice against lipopolysaccharide-induced acute lung injury (48). Indeed, compared to individual therapies, our concurrent treatment with anti-cANGPTL4 MAb and moxifloxacin considerably extended the survival of mice with secondary bacterial pneumonia. Due to the different modes of action compared to antibiotics, immunotherapies with antibodies are highly effective against secondary DRSP infections caused by antibiotic-resistant pathogens. The coimmunoneutralization of cANGPTL4 and pneumolysin was effective against secondary bacterial pneumonia, culminating in improved pulmonary function and longer survival times. Taken together, our study highlights that host-directed therapeutic strategies are valuable adjuncts to complement and enhance standard antimicrobial treatments.

## MATERIALS AND METHODS

### Virus and bacteria.

Influenza virus A/Puerto Rico/8/34 H1N1 strain (PR8) was obtained from the American Type Culture Collection and propagated in embryonated eggs. Viral titers were determined by plaque assay. Serotypes 3 and 19F of S. pneumoniae were derived from clinical isolates in Singapore and cultured in brain heart infusion broth (Sigma 53286) supplemented with 5% heat-inactivated fetal bovine serum (FBS) under anaerobic conditions until the mid-logarithmic phase.

### Animals and infection.

Female BALB/c mice (8 to 12 weeks old) were purchased from InVivos, Singapore, and kept at the biosafety level 2 (BSL-2) animal facility of Nanyang Technological University, Singapore. All animal experiments were approved by the Institutional Animal Care and Use Committee (IACUC) at Nanyang Technological University, Singapore (A0321 and A0200AZ), and National University of Singapore (050/11). Mice were anesthetized using 80 mg/kg body weight ketamine and 10 mg/kg body weight xylazine and infected via intratracheal delivery with the PR8 virus at a sublethal dose of 10 PFU in phosphate-buffered saline (PBS). At day 7 post-influenza infection, 100 CFU of serotype 3 or 5,000 CFU of serotype 19F of S. pneumoniae in PBS was delivered intratracheally to the mice. Sham infections were performed using sterile PBS. Immediately after bacterial infection, mice were injected intraperitoneally with moxifloxacin (100 mg/kg body weight), anti-ANGPTL4 antibody (10 mg/kg body weight), antipneumolysin antibody (10 mg/kg body weight), or control IgG (10 mg/kg body weight) daily starting from day 7 post-influenza infection until harvest. The mice were euthanized at specified time points, and lung tissues were harvested. Lung lobes were either fixed in 4% paraformaldehyde for histologic analysis or snap-frozen in liquid nitrogen and kept at −80°C for subsequent assays.

### Antibodies.

The anti-mouse cANGPTL4 antibody and anti-human cANGPTL4 antibody were produced in-house as described previously (52, 53). The antipneumolysin antibody was prepared by culturing an MG12 hybridoma culture in Dulbecco’s modified Eagle’s medium (DMEM) with 10% FBS and purified from the culture medium using the IgG purification kit (Merck Millipore, no. LSK2ABG20). The anti-CD68 antibody was purchased from Abcam (no. 31630).

### Immunofluorescent staining of lung sections.

Fixed mouse lungs were dehydrated and embedded in paraffin. Five-micrometer sections were mounted on slides coated with Superfrost Plus (Thermal Scientific). Sections were dewaxed with xylene and rehydrated in water. Immunofluorescent staining was performed as previously described (24). Sections were mounted with an antifade reagent containing DAPI (4′,6-diamidino-2-phenylindole [Invitrogen]), and images were captured by Observer Z1 fluorescence microscope (Carl Zeiss). Hematoxylin and eosin (H&E) staining was performed for histopathologic analysis of the lung tissues. S. pneumoniae in the lung sections was detected by employing fluorescent *in situ* hybridization (FISH) as described, with minor modifications (54). Briefly, following the dehydration of fixed lung sections in an ethanol series (3 min each in 50, 80, and 98% ethanol), overnight hybridization to 16S rRNA of S. pneumoniae was performed with an Spn probe tagged with Cy5 in a hybridization buffer containing 30% formamide (55). After the washing steps, the sections were mounted with an antifade reagent and imaged using the LSM780 inverted confocal laser-scanning microscope (Carl Zeiss) fitted with a Plan Apochromat 100×/1.4 oil lens objective, with excitation at 561 nm (autofluorescence) and 633 nm (Cy5). Microscopic images were processed using the Imaris software (version 8.2.0). S. pneumoniae was pseudocolored green.

### Sorting of immune cells.

Immune cells were harvested from influenza virus- and S. pneumoniae serotype 3-coinfected mice at day 9 post-influenza virus infection by bronchoalveolar lavage. One milliliter of sterile PBS was injected into the mouse lung through the trachea and retrieved into the syringe. The BALF contained immune cells and damaged lung tissues. Cells were pelleted by centrifugation at 500 × *g* for 10 min, stained for flow cytometry, and sorted using a BD FACSAria. The following surface markers were used: macrophages (F4/80-fluorescein isothiocyanate [FITC] and CD45-phycorerythrin [PE]), neutrophils (Ly6G-FITC), natural killer cells (NK1.1-PE), cytotoxic T cells (Tc; CD3-allophycocyanin [APC] and CD8-FITC), T-helper cells (Th; CD3-APC and CD4-PE), and B cells (CD19-APC). Unstained cells served as negative controls. Antibodies for cell sorting were from BioLegend. The sorted cells were used for RNA extraction and quantitative real-time reverse transcription-PCR (RT-PCR).

### Quantitative real-time RT-PCR and immunoblotting.

Total RNA was extracted and reverse transcribed as previously described (56, 57). PCR was performed with the Bio-Rad CFX-96 real-time system using KAPA Sybr fast qPCR master mix according to the manufacturer's instructions (KAPA Biosystems). The primers used were mouse ANGPTL4 (forward, 5′-CCCCACGCACCTAGACAATG-3′; reverse, 5′-CCTCCATCTGAAGTCATCTCA-3′) and housekeeping primer RPL27 (forward, 5′-CGCAAAGCTGTCATCGTG-3′; reverse, 5′-CGCAAAGCTGTCATCGTG-3′). Lung tissues were lysed with M-PER mammalian extraction buffer (Pierce), and the protein concentration was determined using the Bio-Rad protein assay kit. Protein samples were heated in 2× Laemmli sample buffer, resolved by SDS-PAGE, and transferred to low-fluorescence polyvinylidene difluoride (PVDF) membranes (Millipore). Immunoblotting was performed as previously described (58).

### RNA sequencing.

Total RNA was extracted from frozen mouse lung tissues using TRIzol (Thermo Fisher). The integrity of the total RNA and the level of DNA contamination were assessed with an Agilent 2100 bioanalyzer (Agilent Technologies) and Qubit 2.0 fluorometer (Invitrogen). Library preparation was performed according to the mRNA-seq sample preparation kit (Illumina) and sequenced using the Illumina MiSeq platform with single-end 75-bp sequencing. Sequencing reads were mapped onto the viral, S. pneumoniae, and mouse genomes using CLC Genomics Workbench 9.0. Differential gene expression was determined using the R statistic package DESeq2 (59). The following criteria were adopted to filter the unique sequence reads: maximum number of hits for a read of 1, minimum length fraction of 0.9, minimum similarity fraction of 0.8, and maximum number of two mismatches. A constant of 1 was added to the raw transcript count value to avoid any problems caused by 0. The DESeq2 package from R/Bioconductor was used for the statistical analysis of the transcript count table. The transcript counts were normalized to the effective library size. The differentially expressed genes were identified by performing a negative binomial test. Transcripts were determined as differentially expressed when they showed a >2-fold change with an adjusted *P* value of <0.05. Pathway analysis and data presentation were performed using Ingenuity Pathway Analysis software (Qiagen Bioinformatics).

### Measurement of cANGPTL4 in BALF and serum from lung disease patients.

BALF samples were obtained from pneumonia patients who fulfilled the ARDS diagnostic criteria of the American European Consensus Conference (60). Controls for BALF samples were obtained from healthy volunteers. BALF was collected with three instillations of sterile physiological saline (50 ml) through a flexible bronchoscope as previously described (61). The collected lavage fluid was passed through two sheets of gauze and centrifuged at 400 × *g* for 10 min at 4°C, and the supernatant was stored at –20°C until use. This study protocol was approved by the Institutional Review Board of Nagasaki University Hospital, Japan. Serum samples were obtained from patients with diagnosed pneumonia before successful treatment with antibiotics from the Biological Resource Center Ferdinand Cabanne (BB-0033-00044; Dijon) and the Centre Hospitalier Universitaire de Dijon, France. Informed consent was obtained from all subjects, and all experiments conformed to the principles outlined in the WMA Declaration of Helsinki and the Department of Health and Human Services Belmont Report.

### Statistical analysis.

Statistical analysis was performed with the unpaired *t* test using GraphPad Prism version 5.03 (GraphPad Software). Values are depicted as mean ± standard error of mean (SEM). A *P* value of <0.05 was considered statistically significant.

### Accession number(s).

RNA sequencing data have been submitted to the NCBI BioProject database under BioProject no. PRJNA394932 and SRA accession no. SRP113049.

## References

[1] Ballinger MN, Standiford TJ 2010 Postinfluenza bacterial pneumonia: host defenses gone awry. J Interferon Cytokine Res 30:643–652. doi:10.1089/jir.2010.0049. 20726789 PMC4367524

[2] Hament J, Kimpen JLL, Fleer A, Wolfs T 1999 Respiratory viral infection predisposing for bacterial disease: a concise review. FEMS Immunol Med Microbiol 26:189–195. doi:10.1111/j.1574-695X.1999.tb01389.x. 10575129

[3] Henriques-Normark B, Normark S 2010 Commensal pathogens, with a focus on Streptococcus pneumoniae, and interactions with the human host. Exp Cell Res 316:1408–1414. doi:10.1016/j.yexcr.2010.03.003. 20227406

[4] Joseph C, Togawa Y, Shindo N 2013 Bacterial and viral infections associated with influenza. Influenza Other Respir Viruses 7(Suppl 2):105–113. doi:10.1111/irv.12089. 24034494 PMC5909385

[5] Kash JC, Taubenberger JK 2015 The role of viral, host, and secondary bacterial factors in influenza pathogenesis. Am J Pathol 185:1528–1536. doi:10.1016/j.ajpath.2014.08.030. 25747532 PMC4450310

[6] Mendes RE, Costello AJ, Jacobs MR, Biek D, Critchley IA, Jones RN 2014 Serotype distribution and antimicrobial susceptibility of USA Streptococcus pneumoniae isolates collected prior to and post introduction of 13-valent pneumococcal conjugate vaccine. Diagn Microbiol Infect Dis 80:19–25. doi:10.1016/j.diagmicrobio.2014.05.020. 24974272

[7] Lamb KE, Flasche S, Diggle M, Inverarity D, Greenhalgh D, Jefferies JM, Smith A, Edwards GFS, Denham B, McMenamin J, McDonald E, Mitchell TJ, Clarke SC, Robertson C 2014 Trends in serotypes and sequence types among cases of invasive pneumococcal disease in Scotland, 1999–2010. Vaccine 32:4356–4363. doi:10.1016/j.vaccine.2013.05.079. 23806244

[8] Shriver Z, Trevejo JM, Sasisekharan R 2015 Antibody-based strategies to prevent and treat influenza. Front Immunol 6:315. doi:10.3389/fimmu.2015.00315. 26217334 PMC4500096

[9] Jackson LA, Janoff EN 2008 Pneumococcal vaccination of elderly adults: new paradigms for protection. Clin Infect Dis 47:1328–1338. doi:10.1086/592691. 18844484 PMC6364558

[10] Tessmer A, Welte T, Martus P, Schnoor M, Marre R, Suttorp N 2009 Impact of intravenous β-lactam/macrolide versus β-lactam monotherapy on mortality in hospitalized patients with community-acquired pneumonia. J Antimicrob Chemother 63:1025–1033. doi:10.1093/jac/dkp088. 19293196

[11] Rodriguez A, Lisboa T, Blot S, Martin-Loeches I, Solé-Violan J, De Mendoza D, Rello J 2009 Mortality in ICU patients with bacterial community-acquired pneumonia: when antibiotics are not enough. Intensive Care Med 35:430–438. doi:10.1007/s00134-008-1363-6. 19066850

[12] García-Suárez MdM, Cima-Cabal MD, Flórez N, García P, Cernuda-Cernuda R, Astudillo A, Vázquez F, De los Toyos JR, Méndez FJ 2004 Protection against pneumococcal pneumonia in mice by monoclonal antibodies to pneumolysin. Infect Immun 72:4534–4540. doi:10.1128/IAI.72.8.4534-4540.2004. 15271913 PMC470670

[13] Orihuela CJ, Gao G, Francis KP, Yu J, Tuomanen EI 2004 Tissue-specific contributions of pneumococcal virulence factors to pathogenesis. J Infect Dis 190:1661–1669. doi:10.1086/424596. 15478073

[14] Lim JH, Stirling B, Derry J, Koga T, Jono H, Woo C-H, Xu H, Bourne P, Ha U-H, Ishinaga H, Xu H, Andalibi A, Feng X-H, Zhu H, Huang Y, Zhang W, Weng X, Yan C, Yin Z, Briles DE, Davis RJ, Flavell RA, Li J-D 2007 Tumor suppressor CYLD regulates acute lung injury in lethal Streptococcus pneumoniae infections. Immunity 27:349–360. doi:10.1016/j.immuni.2007.07.011. 17723219

[15] Albrich WC, Monnet DL, Harbarth S 2004 Antibiotic selection pressure and resistance in Streptococcus pneumoniae and Streptococcus pyogenes. Emerg Infect Dis 10:514–517. doi:10.3201/eid1003.030252. 15109426 PMC3322805

[16] Kim SH, Song J-H, Chung DR, Thamlikitkul V, Yang Y, Wang H, Lu M, So T-K, Hsueh P-R, Yasin RM, Carlos CC, Pham HV, Lalitha MK, Shimono N, Perera J, Shibl AM, Baek JY, Kang C-I, Ko KS, Peck KR 2012 Changing trends in antimicrobial resistance and serotypes of Streptococcus pneumoniae isolates in Asian countries: an Asian Network for Surveillance of Resistant Pathogens (ANSORP) study. Antimicrob Agents Chemother 56:1418–1426. doi:10.1128/AAC.05658-11. 22232285 PMC3294909

[17] Nuermberger EL, Bishai WR 2004 Antibiotic resistance in Streptococcus pneumoniae: what does the future hold? Clin Infect Dis 38(Suppl 4):S363–S371. doi:10.1086/382696. 15127371

[18] Gray BM, Dillon HC 1986 Clinical and epidemiologic studies of pneumococcal infection in children. Pediatr Infect Dis 5:201–207. doi:10.1097/00006454-198603000-00009. 3952010

[19] Clancy CJ, Kalil AC, Fowler VG, Ghedin E, Kolls JK, Nguyen MH 2014 Emerging and resistant infections. Ann Am Thorac Soc 11(Suppl 4):S193–S200. doi:10.1513/AnnalsATS.201402-069PL. 25148425 PMC4200571

[20] Cornick JE, Bentley SD 2012 Streptococcus pneumoniae: the evolution of antimicrobial resistance to beta-lactams, fluoroquinolones and macrolides. Microbes Infect 14:573–583. doi:10.1016/j.micinf.2012.01.012. 22342898

[21] Barcenas-Morales G, Jandus P, Döffinger R 2016 Anticytokine autoantibodies in infection and inflammation: an update. Curr Opin Allergy Clin Immunol 16:523–529. doi:10.1097/ACI.0000000000000316. 27755185

[22] Ricard J-D 2012 New therapies for pneumonia. Curr Opin Pulm Med 18:181–186. doi:10.1097/MCP.0b013e3283520fec. 22388584

[23] Xiao YL, Kash JC, Beres SB, Sheng ZM, Musser JM, Taubenberger JK 2013 High-throughput RNA sequencing of a formalin-fixed, paraffin-embedded autopsy lung tissue sample from the 1918 influenza pandemic. J Pathol 229:535–545. doi:10.1002/path.4145. 23180419 PMC3731037

[24] Li L, Chong HC, Ng SY, Kwok KW, Teo Z, Tan EHP, Choo CC, Seet JE, Choi HW, Buist ML, Chow VTK, Tan NS 2015 Angiopoietin-like 4 increases pulmonary tissue leakiness and damage during influenza pneumonia. Cell Rep 10:654–663. doi:10.1016/j.celrep.2015.01.011. 25660016 PMC7185373

[25] Song J-H, Dagan R, Klugman KP, Fritzell B 2012 The relationship between pneumococcal serotypes and antibiotic resistance. Vaccine 30:2728–2737. doi:10.1016/j.vaccine.2012.01.091. 22330126

[26] Song J-H, Jung S-I, Ko KS, Kim NY, Son JS, Chang H-H, Ki HK, Oh WS, Suh JY, Peck KR, Lee NY, Yang Y, Lu Q, Chongthaleong A, Chiu C-H, Lalitha MK, Perera J, Yee TT, Kumarasinghe G, Jamal F, Kamarulzaman A, Parasakthi N, Van PH, Carlos C, So T, Ng TK, Shibl A 2004 High prevalence of antimicrobial resistance among clinical Streptococcus pneumoniae isolates in Asia (an ANSORP study). Antimicrob Agents Chemother 48:2101–2107. doi:10.1128/AAC.48.6.2101-2107.2004. 15155207 PMC415617

[27] Julkunen I, Mele K, Nyqvist M, Pirhonen J, Sareneva T 2001 Inflammatory responses in influenza A virus infection. Vaccine 19:32–37. 10.1016/s0264-410x(00)00275-9 11163460

[28] McGill J, Heusel JW, Legge KL 2009 Innate immune control and regulation of influenza virus infections. J Leukoc Biol 86:803–812. doi:10.1189/jlb.0509368. 19643736 PMC2752015

[29] Kohlmeier JE, Woodland DL 2009 Immunity to respiratory viruses. Annu Rev Immunol 27:61–82. doi:10.1146/annurev.immunol.021908.132625. 18954284

[30] Julkunen I, Sareneva T, Pirhonen J, Ronni T, Melén K, Matikainen S 2001 Molecular pathogenesis of influenza A virus infection and virus-induced regulation of cytokine gene expression. Cytokine Growth Factor Rev 12:171–180. doi:10.1016/S1359-6101(00)00026-5. 11325600

[31] Nicholls J, Peiris M 2005 Good ACE, bad ACE do battle in lung injury, SARS. Nat Med 11:821–822. doi:10.1038/nm0805-821. 16079870 PMC7095949

[32] Monsalvo AC, Batalle JP, Lopez MF, Krause JC, Klemenc J, Hernandez JZ, Maskin B, Bugna J, Rubinstein C, Aguilar L, Dalurzo L, Libster R, Savy V, Baumeister E, Aguilar L, Cabral G, Font J, Solari L, Weller KP, Johnson J, Echavarria M, Edwards KM, Chappell JD, Crowe JE, Williams JV, Melendi GA, Polack FP 2011 Severe pandemic 2009 H1N1 influenza disease due to pathogenic immune complexes. Nat Med 17:195–199. doi:10.1038/nm.2262. 21131958 PMC3034774

[33] Snelgrove RJ, Edwards L, Rae AJ, Hussell T 2006 An absence of reactive oxygen species improves the resolution of lung influenza infection. Eur J Immunol 36:1364–1373. doi:10.1002/eji.200635977. 16703568

[34] Kobasa D, Jones SM, Shinya K, Kash JC, Copps J, Ebihara H, Hatta Y, Kim JH, Halfmann P, Hatta M, Feldmann F, Alimonti JB, Fernando L, Li Y, Katze MG, Feldmann H, Kawaoka Y 2007 Aberrant innate immune response in lethal infection of macaques with the 1918 influenza virus. Nature 445:319–323. doi:10.1038/nature05495. 17230189

[35] Buchweitz JP, Harkema JR, Kaminski NE 2007 Time-dependent airway epithelial and inflammatory cell responses induced by influenza virus A/PR/8/34 in C57BL/6 mice. Toxicol Pathol 35:424–435. doi:10.1080/01926230701302558. 17487773

[36] Small C, McCormick S, Gill N, Kugathasan K, Santosuosso M, Donaldson N, Heinrichs DE, Ashkar A, Xing Z 2008 NK cells play a critical protective role in host defense against acute extracellular Staphylococcus aureus bacterial infection in the lung. J Immunol 180:5558–5568. doi:10.4049/jimmunol.180.8.5558. 18390740

[37] Small C-L, Shaler CR, McCormick S, Jeyanathan M, Damjanovic D, Brown EG, Arck P, Jordana M, Kaushic C, Ashkar AA, Xing Z 2010 Influenza infection leads to increased susceptibility to subsequent bacterial superinfection by impairing NK cell responses in the lung. J Immunol 184:2048–2056. doi:10.4049/jimmunol.0902772. 20083661

[38] Hartshorn KL 2010 New look at an old problem: bacterial superinfection after influenza. Am J Pathol 176:536–539. doi:10.2353/ajpath.2010.090880. 20019194 PMC2808060

[39] Uchide N, Toyoda H 2011 Antioxidant therapy as a potential approach to severe influenza-associated complications. Molecules 16:2032–2052. doi:10.3390/molecules16032032. 21358592 PMC6259602

[40] Aldridge JR, Moseley CE, Boltz DA, Negovetich NJ, Reynolds C, Franks J, Brown SA, Doherty PC, Webster RG, Thomas PG 2009 TNF/iNOS-producing dendritic cells are the necessary evil of lethal influenza virus infection. Proc Natl Acad Sci U S A 106:5306–5311. doi:10.1073/pnas.0900655106. 19279209 PMC2664048

[41] Moorthy AN, Rai P, Jiao H, Wang S, Tan KB, Qin L, Watanabe H, Zhang Y, Teluguakula N, Chow VTK 2016 Capsules of virulent pneumococcal serotypes enhance formation of neutrophil extracellular traps during in vivo pathogenesis of pneumonia. Oncotarget 7:19327–19340. doi:10.18632/oncotarget.8451. 27034012 PMC4991386

[42] Narayana Moorthy A, Narasaraju T, Rai P, Perumalsamy R, Tan KB, Wang S, Engelward B, Chow VTK 2013 In vivo and in vitro studies on the roles of neutrophil extracellular traps during secondary pneumococcal pneumonia after primary pulmonary influenza infection. Front Immunol 4:56. doi:10.3389/fimmu.2013.00056. 23467809 PMC3587798

[43] Jerebtsova M, Nekhai S 2015 Therapeutics for postexposure treatment of Ebola virus infection. Future Virol 10:221–232. doi:10.2217/fvl.14.109. 26213559 PMC4508675

[44] Murray J, Saxena S, Sharland M 2014 Preventing severe respiratory syncytial virus disease: passive, active immunisation and new antivirals. Arch Dis Child 99:469–473. doi:10.1136/archdischild-2013-303764. 24464977

[45] Pelegrin M, Naranjo-Gomez M, Piechaczyk M 2015 Antiviral monoclonal antibodies: can they be more than simple neutralizing agents? Trends Microbiol 23:653–665. doi:10.1016/j.tim.2015.07.005. 26433697 PMC7127033

[46] Mitchell TJ, Mendez F, Paton JC, Andrew PW, Boulnois GJ 1990 Comparison of pneumolysin genes and proteins from Streptococcus pneumoniae types 1 and 2. Nucleic Acids Res 18:4010. doi:10.1093/nar/18.13.4010. 2374733 PMC331126

[47] Li L, Chow VTK, Tan NS 2015 Targeting vascular leakage in lung inflammation. Oncotarget 6:19338–19339. doi:10.18632/oncotarget.4907. 26305720 PMC4637277

[48] Guo L, Li S, Zhao Y, Qian P, Ji F, Qian L, Wu X, Qian G 2015 Silencing angiopoietin-like protein 4 (ANGPTL4) protects against lipopolysaccharide-induced acute lung injury via regulating SIRT1/NF-kB pathway. J Cell Physiol 230:2390–2402. doi:10.1002/jcp.24969. 25727991

[49] Sugiyama MG, Armstrong SM, Wang C, Hwang D, Leong-Poi H, Advani A, Advani S, Zhang H, Szaszi K, Tabuchi A, Kuebler WM, Van Slyke P, Dumont DJ, Lee WL 2015 The Tie2-agonist Vasculotide rescues mice from influenza virus infection. Sci Rep 5:11030. doi:10.1038/srep11030. 26046800 PMC4457136

[50] Ito Y, Oike Y, Yasunaga K, Hamada K, Miyata K, Matsumoto S, Sugano S, Tanihara H, Masuho Y, Suda T 2003 Inhibition of angiogenesis and vascular leakiness by angiopoietin-related protein 4. Cancer Res 63:6651–6657. 14583458

[51] Huang R-L, Teo Z, Chong HC, Zhu P, Tan MJ, Tan CK, Lam CRI, Sng MK, Leong DTW, Tan SM, Kersten S, Ding JL, Li HY, Tan NS 2011 ANGPTL4 modulates vascular junction integrity by integrin signaling and disruption of intercellular VE-cadherin and claudin-5 clusters. Blood 118:3990–4002. doi:10.1182/blood-2011-01-328716. 21841165

[52] Goh YY, Pal M, Chong HC, Zhu P, Tan MJ, Punugu L, Tan CK, Huang R-L, Sze SK, Tang MBY, Ding JL, Kersten S, Tan NS 2010 Angiopoietin-like 4 interacts with matrix proteins to modulate wound healing. J Biol Chem 285:32999–33009. doi:10.1074/jbc.M110.108175. 20729546 PMC2963335

[53] Zhu P, Tan MJ, Huang RL, Tan CK, Chong HC, Pal M, Lam CRI, Boukamp P, Pan JY, Tan SH, Kersten S, Li HY, Ding JL, Tan NS 2011 Angiopoietin-like 4 protein elevates the prosurvival intracellular O_2_^−^:H_2_O_2_ ratio and confers anoikis resistance to tumors. Cancer Cell 19:401–415. doi:10.1016/j.ccr.2011.01.018. 21397862

[54] Quast C, Pruesse E, Yilmaz P, Gerken J, Schweer T, Yarza P, Peplies J, Glöckner FO 2013 The SILVA ribosomal RNA gene database project: improved data processing and web-based tools. Nucleic Acids Res 41:D590–D596. doi:10.1093/nar/gks1219. 23193283 PMC3531112

[55] Kempf VA, Trebesius K, Autenrieth IB 2000 Fluorescent In situ hybridization allows rapid identification of microorganisms in blood cultures. J Clin Microbiol 38:830–838. 10655393 10.1128/jcm.38.2.830-838.2000 PMC86216

[56] Lam CRI, Tan MJ, Tan SH, Tang MBY, Cheung PCF, Tan NS 2011 TAK1 regulates SCF expression to modulate PKBα activity that protects keratinocytes from ROS-induced apoptosis. Cell Death Differ 18:1120–1129. doi:10.1038/cdd.2010.182. 21233843 PMC3131962

[57] Lam CRI, Tan C, Teo Z, Tay CY, Phua T, Wu YL, Cai PQ, Tan LP, Chen X, Zhu P, Tan NS 2013 Loss of TAK1 increases cell traction force in a ROS-dependent manner to drive epithelial-mesenchymal transition of cancer cells. Cell Death Dis 4:e848. doi:10.1038/cddis.2013.339. 24113182 PMC3824649

[58] Chong HC, Chan JSK, Goh CQ, Gounko NV, Luo B, Wang X, Foo S, Wong MTC, Choong C, Kersten S, Tan NS 2014 Angiopoietin-like 4 stimulates STAT3-mediated iNOS expression and enhances angiogenesis to accelerate wound healing in diabetic mice. Mol Ther 22:1593–1604. doi:10.1038/mt.2014.102. 24903577 PMC4435481

[59] Love MI, Huber W, Anders S 2014 Moderated estimation of fold change and dispersion for RNA-seq data with DESeq2. Genome Biol 15:550. doi:10.1186/s13059-014-0550-8. 25516281 PMC4302049

[60] Bernard GR, Artigas A, Brigham KL, Carlet J, Falke K, Hudson L, Lamy M, Legall JR, Morris A, Spragg R 1994 The American-European Consensus Conference on ARDS. Definitions, mechanisms, relevant outcomes, and clinical trial coordination. Am J Respir Crit Care Med 149:818–824. doi:10.1164/ajrccm.149.3.7509706. 7509706

[61] Sakamoto N, Kakugawa T, Hara A, Nakashima S, Yura H, Harada T, Ishimoto H, Yatera K, Kuwatsuka Y, Hara T, Ichinose K, Obase Y, Ishimatsu Y, Kohno S, Mukae H 2015 Association of elevated α-defensin levels with interstitial pneumonia in patients with systemic sclerosis. Respir Res 16:148. doi:10.1186/s12931-015-0308-1. 26654954 PMC4676113

