# Supplementary figures and images for: Antibody Treatment against Angiopoietin-Like 4 Reduces Pulmonary Edema and Injury in Secondary Pneumococcal Pneumonia
Source: mBio. 2019 Jun 4;10(3):e02469-18. doi: 10.1128/mBio.02469-18 (PMC6550533; doi:10.1128/mBio.02469-18)

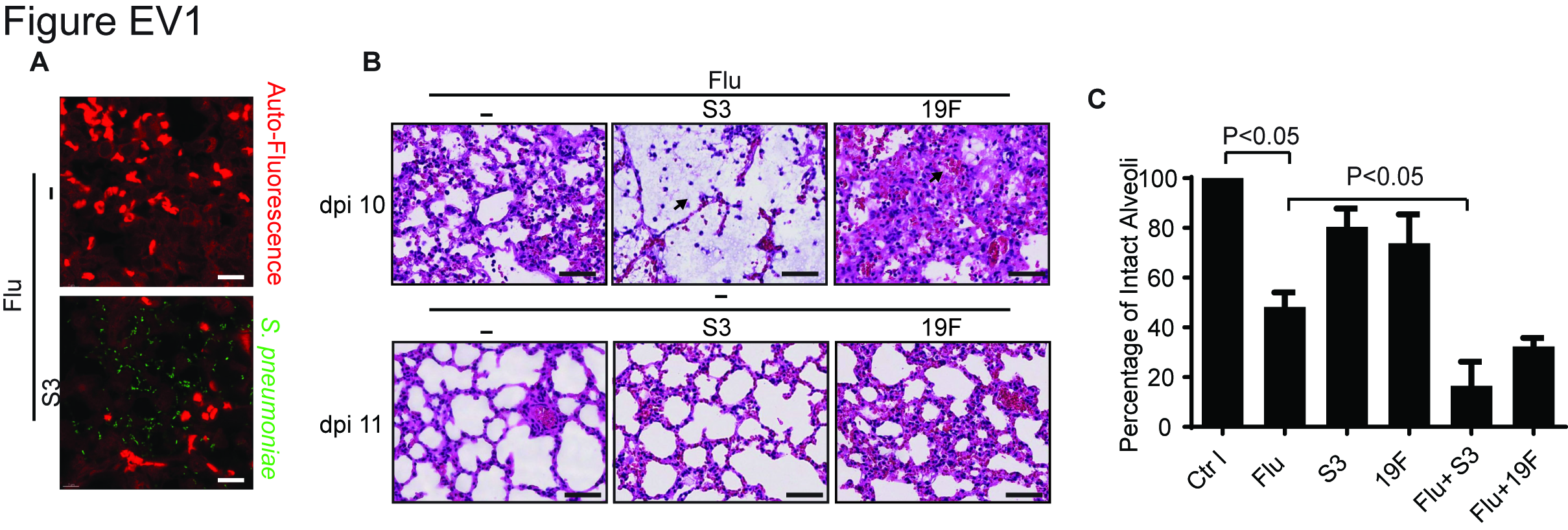

Supplement: FIG S1 [file mBio.02469-18-sf001.tif]

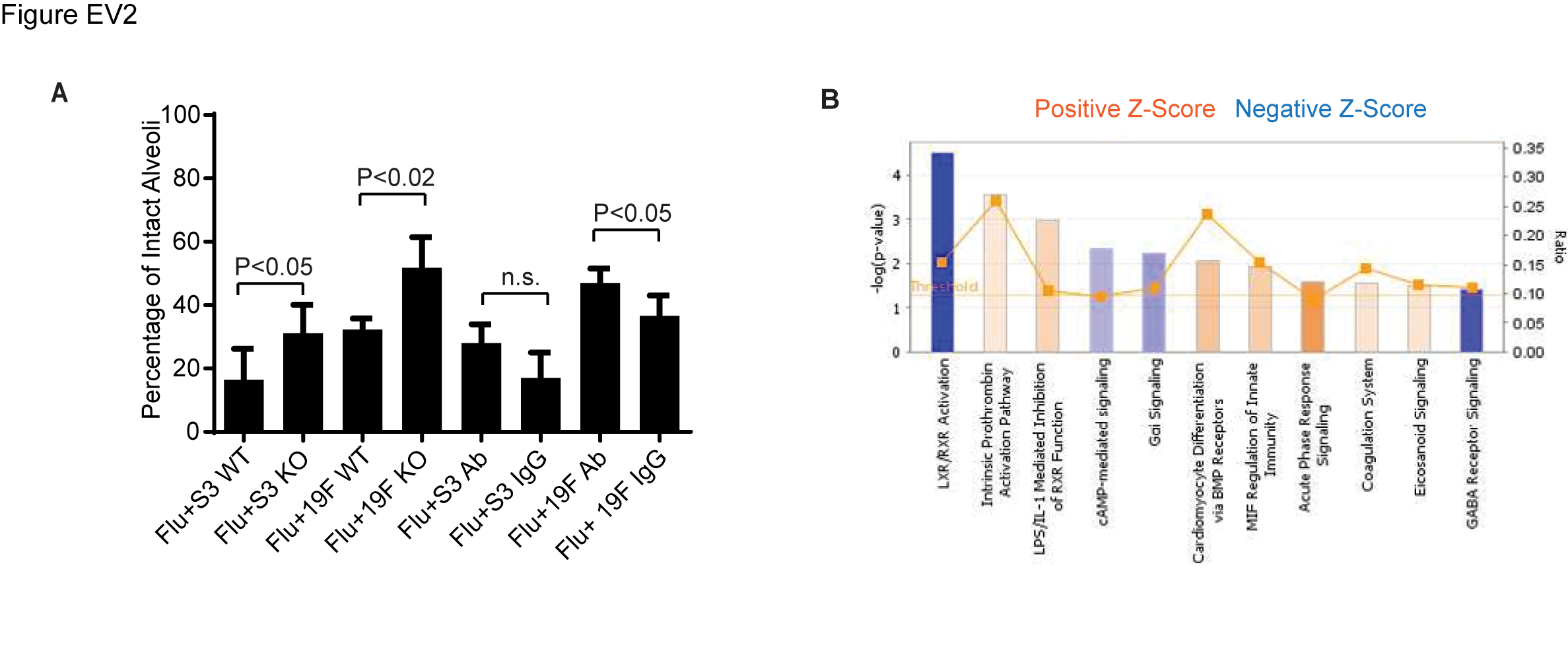

Supplement: FIG S2 [file mBio.02469-18-sf002.tif]

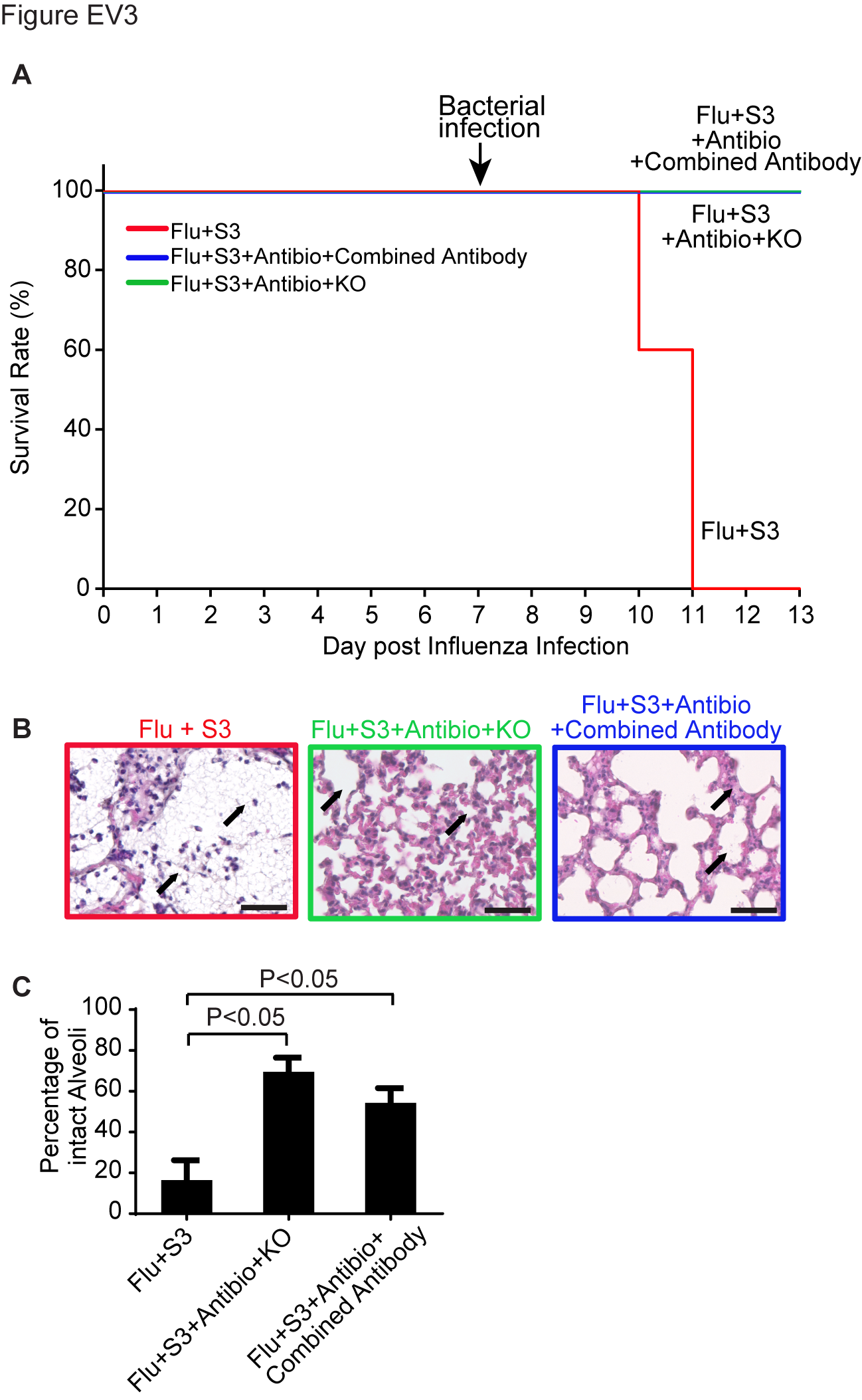

Supplement: FIG S3 [file mBio.02469-18-sf003.tif]
